# Coeliac Disease

**DOI:** 10.1155/2014/623784

**Published:** 2014-05-28

**Authors:** Raffaella Nenna, Stefano Guandalini, Alina Popp, Kalle Kurppa

**Affiliations:** ^1^Department of Paediatrics, “Sapienza” University of Rome, Italy; ^2^Section of Gastroenterology, Department of Pediatrics, University of Chicago, Chicago, IL, USA; ^3^University of Medicine and Pharmacy “Carol Davila”, Bucharest, Romania; ^4^Tampere Center for Child Health Research, University of Tampere and Tampere University Hospital, Finland

Celiac disease is an autoimmune disorder, caused by a permanent intolerance to gluten contained in wheat and to similar prolamines present in barley and rye. It is a common disease as its prevalence, in Caucasian populations, is about 1%. There are several patterns of clinical presentation: typical (with gastrointestinal symptoms), atypical (extraintestinal manifestations), and silent forms, all characterized by typical histological lesions in the small bowel mucosa. Nowadays the gluten-free diet for life is the only therapy available for CD. Considerable progress has been made due to advances in molecular biology, which have allowed better understanding of the genetic mechanisms involved in CD and the identification of new pathogenetic pathways that have the potential to be targeted by new drugs. In this special issue, we have invited authors to contribute original papers that will stimulate the continuing efforts to understand the pathogenesis and heterogeneous clinical presentation of celiac disease.

Immunogenic peptides, created by deamidation of food-derived gliadin peptides by small intestinal tissue transglutaminase, are presented by antigen-presenting cells, mostly dendritic cells bearing HLA-DQ2 and DQ8 molecules, to proinflammatory CD4+ T cells, activating them. E. Liu et al. investigated peripheral T cell responses from young children with newly diagnosed CD prior to treatment with a gluten-free diet, finding that T cell reactivity is heterogeneous but favors reactivity with *α*-gliadin epitopes more than *γ*-gliadin.

Metabolomics is a rapidly emerging new concept in science that appears to have relevant application also in the field of celiac disease. In their meticulous review, A. Calabrò et al. highlight the metabolomics perspective on celiac disease. Furthermore, the growing recognition of the important role of gut microbiota in the development of celiac disease and other food-related disorders is addressed and thoroughly reviewed.

Healing of the small bowel mucosa is directly dependent on the adherence to the gluten-free diet, and its assessment by noninvasive tools has been a long-time objective of research in celiac disease, with the aim of establishing suitable minimally invasive methods both for diagnosing and for adequate follow-up of celiac disease and of the adequacy of the gluten-free diet. In their study, E. Trigoni et al. concluded that anti-endomysium antibodies had better ability, at least in adult individuals, than anti-tissue transglutaminase to predict celiac disease and to assess the gluten-free diet in the critical period of the first semester after diagnosis and the beginning of the diet.

Among the atypical clinical forms of CD, oral signs are often neglected but could offer important diagnostic clues in challenging diagnoses. Lesions in the oral mucosa or defects in dental enamel can in fact be present in celiac patients. The paper of M. Erriu et al., evaluating oral signs and different DQ2 haplotypes, highlights the fundamental role that dentists can play in early recognition of CD.

It is well known that untreated celiac disease patients develop specific serum autoantibodies against transglutaminase 2. Recently it has been discovered that corresponding circulating antibodies against transglutaminase 6 (TG6) may play a role in particular gluten-related neurological disorders. In their study, R. Stenberg et al. demonstrate that the presence of anti-TG6 antibodies is increased in subjects with early neurological injury resulting in cerebral palsy, suggesting that an early brain insult may lead to subsequent TG6 autoimmunity.

Due to their common genetic background, celiac disease, type 1 diabetes mellitus, and autoimmune thyroiditis are often associated. M. van der Pals et al. confirmed three times higher prevalence of thyroid autoimmunity markers (autoantibodies against thyroid peroxidase) in celiac disease children compared with 12-year-old healthy controls but no difference in thyroid autoimmunity between clinically detected and screen-detected celiac disease children.

*Raffaella Nenna* *Stefano Guandalini* *Alina Popp* *Kalle Kurppa*

